# Cost-effectiveness of a universal strategy of brief dietary intervention for primary prevention in primary care: population-based cohort study and Markov model

**DOI:** 10.1186/1478-7547-12-4

**Published:** 2014-02-02

**Authors:** Martin C Gulliford, Nawaraj Bhattarai, Judith Charlton, Caroline Rudisill

**Affiliations:** 1 King’s College London, Department of Primary Care and Public Health Sciences, Capital House, 42 Weston St, London SE1 3QD, UK; 2 Department of Social Policy, London School of Economics and Political Science, London, UK

**Keywords:** Dietary intervention, Primary care, Markov model, Cost effectiveness, Outcomes, Diabetes, Coronary heart disease, Stroke, Colorectal cancer, Depression

## Abstract

**Background:**

A healthy diet is associated with reduced risk of diabetes, cardiovascular disease and cancer. The study aimed to evaluate the cost-effectiveness of a universal strategy to promote healthy diet through brief intervention in primary care.

**Methods:**

The research was informed by a systematic review of randomised trials which found that brief interventions in primary care may be associated with a 0.5 portion per day increase in fruit and vegetable consumption. A Markov model that included five long-term conditions (diabetes, coronary heart disease, stroke, colorectal cancer and depression) was developed. Empirical data from a large cohort of United Kingdom-based participants sampled from the Clinical Practice Research Datalink populated the model. Simulations compared an intervention promoting healthy diet over 5 years in healthy adults, and standard care in which there was no intervention. The annual cost of intervention, in the base case, was one family practice consultation per participant year. Health service costs were included and the model adopted a lifetime perspective. The primary outcome was net health benefit in quality adjusted life years (QALYs).

**Results:**

A cohort of 262,704 healthy participants entered the model. Intervention was associated with an increase in life years lived free from physical disease of 41.9 (95% confidence interval -17.4 to 101.0) per 1,000 participants entering the model (probability of increase 88.0%). New incidences of disease states were reduced by 28.4 (18.7 to 75.8) per 1,000, probability reduced 84.6%. Discounted incremental QALYs were 4.3 (-8.8 to 18.0) per 1,000, while incremental costs were £139,755 (£60,466 to 220,059) per 1,000. Net health benefits at £30,000 per QALY were -0.32 (-13.8 to 13.5) QALYs per 1,000 participants (probability cost-effective 47.9%). When the intervention was restricted to adults aged 50 to 74 years, net health benefits were 2.94 (-21.3 to 26.4) QALYs per 1000, probability increased 59.0%.

**Conclusions:**

A universal strategy to promote healthy diet through brief intervention in primary care is unlikely to be cost-effective, even when delivered at low unit cost. A targeted strategy aimed at older individuals at higher risk of disease might be more cost-effective. More effective dietary change interventions are needed.

## Background

Diet is an important determinant of health [1]. Dietary intakes are associated with the risk of chronic diseases including diabetes, cardiovascular diseases and cancer [2]. Dietary risk factors, including low consumption of fruit and vegetables, together with physical inactivity, account for about 10% of the total global burden of disease [1]. Dietary exposures are important across the life-course and present dietary habits may determine the development of chronic diseases in later life [3]. It is estimated that a long term increase in intake of fruit and vegetables of one portion per day (80 g/day) might be associated with a 10% relative reduction in risk of ischaemic heart disease and 6% reduction in stroke, with between 1% and 6% reduction in risk of certain cancers [4]. National recommendations advocate increasing the consumption of a variety of fruit and vegetables each day in order to reduce risks of obesity, diabetes, cardiovascular disease and certain cancers [5].

The regularity of patient consultations, and the value patients place on primary care advice, offer primary care clinicians an opportunity to promote healthy lifestyles, including dietary change [6]. A number of randomised trials have evaluated the effectiveness of dietary interventions for primary prevention in primary care [7-16]. These have shown small beneficial effects on dietary intakes including consumption of fruits, vegetables, fibre, fat and on serum cholesterol, with benefits maintained over a period of up to one year. However, in a meta-analysis of trials in primary care settings, we found that the mean increment in fruit and vegetable consumption associated with brief interventions in primary care amounted to about 0.5 (95% confidence interval 0.13 to 0.87) portions per participant per day [17]. This is consistent with the findings of Rees et al. [18], which included a wider range of intervention settings and participants in their review. An increase in fruit and vegetable consumption of this magnitude might yield worthwhile health benefits, if it were to be maintained over long enough periods of time or in a large enough population. However, primary research studies of sufficient scale and duration have not yet been implemented.

The present research used a Markov simulation model to evaluate the potential long-term outcomes and costs of a universal strategy to promote healthy diet through brief intervention in primary care. The research specifically aimed to evaluate the cost-effectiveness of a universal strategy of brief dietary intervention for primary prevention in primary care. The target population for the research was the general population of healthy adults, free from chronic disease, registered in primary care. We compared a strategy of brief intervention to promote healthy diet delivered to all healthy adults, focusing on increasing consumption of fruit and vegetables, with ‘standard care’ in which there is no systematic approach to dietary intervention.

## Methods

We utilised a similar methodological approach to one reported previously to evaluate brief interventions to promote physical activity [19].

### Overview and Markov model

A Markov model was designed drawing on previously reported research [20]. Participants entering the model were aged 30 years and over because of the low risk of chronic disease at younger ages. Healthy participants free from chronic disease, referred to as At Risk, could develop one of the disease states of interest including Diabetes mellitus, Coronary Heart Disease, Stroke or Colorectal cancer. Participants were allowed to progress to multiple disease states, representing all potential combinations of the selected conditions, consistent with the frequent development of multiple morbidity in primary care [21]. Participants in each state were allowed to progress to Depression, or to remain Not Depressed [22]. Depression was included in the Model because the prevalence of depression is increased in many chronic diseases and depression is associated with higher health care costs [22]. Depression was considered to be reversible; in each cycle participants transitioned to Depression on a probabilistic basis, based on the prevalence of depression observed in that state. All model states might progress to death. The model included a total of 32 states, representing the potential combinations of included diseases and depression. A schematic diagram of the model was previously reported [19]. The cost analysis was from the health care perspective and only costs of health care utilisation were included. A life time horizon was used for the analysis. The model was stratified by gender and single year of age.

### CPRD cohort and empirical inputs to the model

The model was populated with empirical estimates drawn from a large cohort of participants registered with family practices participating in the UK Clinical Practice Research Datalink (CPRD), previously known as the General Practice Research Database (GPRD). Participants were sampled from family practices continuously contributing data to the CPRD between 1 January 2004 and 30 October 2010. Participants comprised a random sample of 299,912 registered patients, aged 30 to 100 years. CPRD data were analysed to estimate, for each state in the model, the incidence of the state, the mortality in each state, the prevalence of depression, and the costs of health care utilisation. Estimates were obtained by 10-year age group and sex. The methodological approach to analysis has been reported previously [22]. Unit costs of health care utilisation were obtained from standard reference sources for 2010 [23]. The Multilex drug code for each prescription record in CPRD was linked to unit costs in the First DataBank Europe database in order to obtain prescription costs [24]. The empirical mean and standard deviation for costs associated with each participant was estimated according to 10-year age group, sex and model state. Utilities for each state were obtained from data published in a compendium of values [25]. Utility values for each state were stratified by single year of age but were the same for men and women.

### Model estimates

The Markov model, with probabilistic cohort simulation, was programmed using R software [26]. Outcomes and costs were compared for Intervention and Standard Care over 70 annual cycles, this allowed the entire cohort to progress either to death or to reach age 100 and exit the model. Annual transition probabilities for the model were obtained by sampling from the beta-binomial distribution, using CPRD data as inputs. Utility values were sampled from the beta distribution. The costs of each state were sampled from the gamma distribution with the mean value from CPRD, by10-year age group, sex, condition and depression status, as the empirical input. The model was implemented with a half cycle correction for the estimation of QALYs and costs.

Total costs and quality adjusted life years (QALYs) were obtained by summing across the 70 cycles of the model included in each simulation. There were 2,000 simulations run for each of intervention or standard care scenarios. Results are expressed as rates per 1,000 healthy participants entering the model. Mean costs, and the 95% range, were obtained from the data for 2,000 simulations. Incremental costs and QALYs were obtained as the difference between intervention and standard care scenarios. Costs and QALYs were discounted using a rate of 3.5%, but QALYs were also discounted at a rate of 1.5% as a sensitivity analysis. Not all simulations were associated with positive incremental costs and QALYs, making the incremental cost-effectiveness ratio a less suitable measure for analysis [27]. Net health benefits (NHB), at a threshold value of £30,000 per QALY, were therefore calculated as the difference between the increment in QALYs and the increment in costs divided by the threshold value of cost per QALY [28]. (pages 128-130) A cost-effectiveness acceptability curve was plotted using a range of threshold values.

### Intervention effects and costs

The intervention was assumed to modify only the incidence of physical disease in healthy participants at risk. Changes in occurrence of multi-disease states, prevalence of depression and mortality were assumed to be secondary to changes in the incidence of diabetes, coronary heart disease, stroke and colorectal cancer. Relative risks of diabetes, coronary heart disease, stroke and colorectal cancer, associated with fruit and vegetable consumption were calculated using two sources of information. The first was a systematic review of randomised trials in primary care [17] that provided an estimated 0.5 (95% confidence interval 0.13 to 0.87) portion per day increment in fruit and vegetable consumption from brief dietary intervention in primary care. This was combined with estimated age-specific relative risks of disease outcomes associated with dietary change derived from the World Health Organization report. (Table 9.28, page 696). The intervention and the intervention effect were modelled to be maintained for five years. In the absence of evidence for the time course of intervention effects, a constant intervention effect was modelled in each of the first five cycles of the model. The cost of the intervention was modelled as a fixed cost per person per year and was assumed initially to be equivalent as the cost of one family practice consultation. Sensitivity analyses was carried out taking costs of intervention equivalent to two family practice consultations per year and 20% of a single family practice consultation per year. In secondary analyses, the effect of intervention was also modelled in selected age groups to allow the comparison of cost-effectiveness between age groups. There were 1,000 simulations performed in each age group because of the greater computational burden.

### Consent

We used anonymous data approved by CPRD without identifying the patient and written informed consent was not required from the patients for the publication of this paper and any accompanying images.

## Results

Table 1 presents the estimated relative risk values for intervention effects on incidence of study conditions. The figures are mean and range of values for 2,000 simulations based on the mean values across all ages in the first cycle of each simulation. An intervention effect of 0.95 indicates a 5% lower incidence rate compared to incidence rate with no intervention. In general, intervention was associated with relative risk reductions of 4% or smaller, when averaged across all age groups.

**Table 1:** Summary of the modelled effect of intervention on incidence of study conditions

**Disease condition**	**Effect of intervention (Relative risk)**	
	**Male**	**Female**
**Diabetes mellitus**	0.965 (0.961 to 0.968)	0.964 (0.961 to 0.968)
**Coronary heart disease**	0.959 (0.955 to 0.963)	0.959 (0.955 to 0.963)
**Stroke**	0.975 (0.972 to 0.978)	0.975 (0.972 to 0.978)
**Colorectal cancer**	0.997 (0.995 to 0.998)	0.997 (0.995 to 0.998)

Figures represent the mean relative risk value (95% interval) for 2,000 simulations, based on the mean values across all ages in the first cycle.

There were 262,704 healthy participants, based on the age distribution of healthy participants aged 30 years and older in CPRD, who entered the model in each simulation (Table 2). There were 129,396 men (mean age 51 years) and 133,308 women (mean age 54 years). Crude incidence rates from the Model under standard care for the age range 30 to 100 years were diabetes 6.5 per 1,000, coronary heart disease 7.6 per 1,000, stroke 4.1 per 1,000 and colorectal cancer 1.0 per 1,000, consistent with empirical values previously reported from analysis of CPRD [29-31]. Following intervention, with the intervention effect continuing for 5 years, there was an increase in life years lived without physical disease of 41.9 (-17.4 to 101.0) per 1,000 participants entering the model. There was 88% probability that life years free from physical disease were increased. There was a modest reduction in new occurrences of diabetes mellitus and coronary heart disease amounting to about 0.5 cases per 1,000 participants entering the model but there was negligible change in new occurrences of stroke and colorectal cancer. Over the course of the model, the number of life years lived with a single physical morbidity was reduced by 28.4 life years per 1,000 participants entering the model, with the probability of reduction of 84.6%. There was some evidence of a reduction in life years with dual morbidities but negligible effect on triple or quadruple morbidities. There was weak evidence of a reduction in overall life years lived with depression, which was consequent on the reduction in morbidity as there was no modelled direct effect of intervention on depression prevalence.

**Table 2:** Health outcomes and cost-effectiveness of a healthy eating intervention in a population of 262,704 healthy participants

	**Difference**	**Probability**
	**(Intervention-standard care)**	**(%)**
Number entering intervention	262,704	
Life years lived without disease (per 1,000)^a^	41.9 (-17.4 to 101.0)	88.0
New incidences per 1,000 of:		
Diabetes mellitus	-0.5 (-2.2 to 1.23)	67.7
Coronary heart disease	-0.6 (-2.4 to 1.3)	69.0
Stroke	0.02 (-1.5 to 1.6)	49.7
Colorectal cancer	0.04 (-0.7 to 0.8)	46.2
Life years lived with physical morbidity (per 1,000)^a^		
Single condition	-28.4 (-75.8 to 18.7)	84.6
Dual conditions	-7.2 (-28.9 to 14.4)	71.0
Triple conditions	-0.7 (-7.5 to 6.1)	56.1
Quadruple conditions	-0.0 (-1.4 to 1.5)	52.2
Life years lived with depression (per 1,000)^a^	-2.9 (-16.6 to 11.2)	63.9
Total life years (per 1,000)^a^	5.7 (-36.6 to 47.3)	41.1
Total intervention costs (£ per 1,000)	153,521 (153,462 to 153,583)	100.0
Incremental costs of non-intervention health care utilisation (£ per 1,000)	-13,765 (-93,093 to 66,556)	38.8
Incremental total costs (£ per 1,000)^a,b^	139,755 (60,466 to 220,059)	99.8
Incremental QALYs (discounted 3.5%) (per 1,000)	4.3 (-8.8 to 18.0)	68.8
Incremental QALYs (discounted 1.5%) (per 1,000)	6.3 (-15.6 to 28.4)	67.0
Net health benefits (QALYs per 1,000)^b,c^	-0.32 (-13.8 to 13.5)	47.9
Probability cost effective	47.9	
At £30,000 per QALY (%)		

^a^per 1,000 healthy participants entering model; ^b^discounted at 3.5%; ^c^net health benefit at a threshold of £30,000 per QALY.

Figures represent mean and 95% range of 2,000 simulations.

The mean discounted incremental costs associated with intervention were £139,755 per 1,000 participants entering the model (Table 2). The mean cost associated with intervention was £153,521 per 1,000 but this was offset by a small decrease in the costs of non-intervention health care utilisation amounting to -£13,765 per 1,000. The mean discounted incremental QALYs associated with intervention were 4.3 per 1,000 participants entering the model. At a threshold of £30,000 per QALY, the mean net health benefit was -0.32 QALYs per 1,000 participants. The probability of intervention being cost effective at cost effectiveness threshold of £30,000 per QALY was 47.9%.

Sensitivity analyses were carried out to explore the effects of varying the discount rate, varying the unit costs of the intervention and varying the age group targeted for intervention. When QALYs were discounted at 1.5%, the mean increment associated with intervention was 6.3 QALYs per 1,000 (Table 2). The mean net benefits at a threshold of £30,000 per QALY were then 1.7 per 1,000, with a probability cost-effective of 54.5%. As expected, the effect of increasing the unit costs of intervention costs was to lower the estimated cost effectiveness (Table 3). If the unit cost of intervention amounted to two family practice consultations per year then mean net health benefits were negative (-5.4, 95% CI -18.9 to 8.4) with the probability of the intervention being cost-effective was 26%. If the cost of the intervention was equivalent to 20% of cost of one family practice consultations per year, equivalent to two minutes out of a 10 minute consultation, then the net health benefit was 3.8 (-9.7 to 17.6) QALYs per 1000, with a probability cost-effective of 66.7%.

**Table 3:** Effect of varying the unit cost of intervention

	**Annual unit cost of intervention**		
	**0.2 GP consultations per year (£7 per participant year)**	**Cost equivalent to one GP consultation per year (£35 per participant year)**	**Cost equivalent to two GP consultations per year (£70 per participant year)**
Incremental QALYs^a^	4.3 (-8.8 to 18.0)	4.3 (-8.8 to 18.0)	4.3 (-8.8 to 18.0)
Incremental cost^a^	16,939	139,755	293,276
	(-62,385 to 97,256)	(60,446 to 220,059)	(213,985 to 373,563)
Net health benefit^a,b^	3.8	-0.32	-5.4
	(-9.7 to 17.6)	(-13.8 to 13.5)	(-18.9 to 8.4)
Probability cost-effective	66.7	47.9	25.6

^**a**^discounted at 3.5%; ^b^net health benefit at a threshold of £30,000 per QALY.

Figures are expressed per 1,000 participants entering the model and represent mean and 95% range of 2,000 simulations.

**Figure 1:**
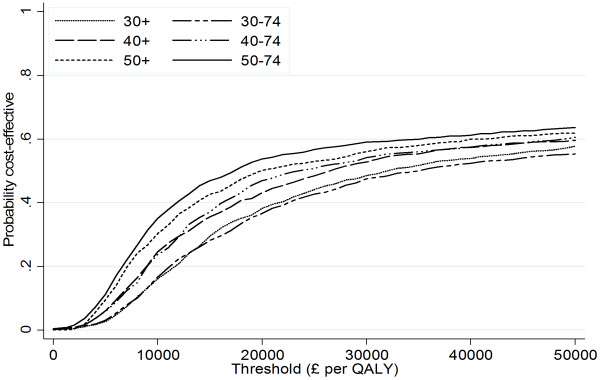
Cost-effectiveness acceptability curves for dietary intervention targeted at selected age groups.

Table 4 and Figure 1 present estimates for the cost effectiveness of a brief dietary intervention in primary care when delivered only to selected age groups. The effect of intervention on fruit and vegetable consumption was assumed to be independent of age, but changes in disease risk from dietary change were age-dependent [3]. Net health benefits at a threshold of £30,000 per QALY were lowest when a universal policy targeting all adults aged greater than 30 years was employed. As the lower age limit for eligibility for intervention was raised, first to 40 years and then to 50 years, then gain in QALYs associated with intervention increased, while the incremental costs per 1,000 participants showed little change. Consequently, net health benefits were higher when the lower age limit for intervention was 50 years, than when intervention included all those over 30 years. The effect of reducing the upper age limit for intervention to 74 years, generally had a more limited impact on the estimated cost-effectiveness of intervention. Even in the most favourable scenario, where the eligibility for intervention was restricted to the age range 50 to 74 years, the probability of the intervention being cost-effective was less than 60% at a threshold of £30,000 per QALY, with little impact from increasing the threshold value (Figure 1).

**Table 4:** Cost-effectiveness of dietary intervention in selected age-groups

**Age group**	**Life years healthy**	**Incremental QALYs**^**a**^	**Incremental Costs**^**a**^	**Net health benefit**^**b**^	**Probability cost-effective (%)**
30 years and over	44.1	4.40	140,716	-0.29	48.5
	(-21.0 to 105.7)	(-10.0 to 18.6)	(63,609 to 217,580)	(-14.5 to 13.6)	
30 to 74 years	42.1	3.84	140,758	-0.85	47.5
	(-23.3 to 107.0)	(-11.2 to 19.1)	(59,557 to 221,842)	(-16.4 to 14.1)	
40 years and over	49.7	5.3	131,152	0.95	52.8
	(-15.8 to 117.6)	(-10.3 to 22.2)	(53,690 to 206,149)	(-14.8 to 17.2)	
40 to 74 years	51.9	5.7	136,606	1.12	54.2
	(-16.3 to 127.7)	(-11.2 to 22.4)	(56,301 to 215,257)	(-16.0 to 17.8)	
50 years and over	55.6	6.8	131,812	2.41	56.0
	(-11.7 to 126.5)	(-14.6 to 28.9)	(60,978 to 208,546)	(-18.6 to 24.6)	
50 to 74 years	61.5	7.4	132,929	2.94	59.0
	(-21.0 to 146.2)	(-16.7 to 31.9)	(56,612 to 205,664)	(-21.3 to 26.4)	

^a^discounted at 3.5%; ^b^net health benefit at a threshold of £30,000 per QALY.

Figures are expressed per 1,000 participants entering the model and represent the mean and 95% range of 1,000 simulations in each age group.

## Discussion

### What this study shows

Our study modelled the health outcomes of a universal strategy for brief intervention to promote dietary change in primary care. We used a very large empirical population registered in UK primary care practices to represent the study population. The intervention effect was derived from a meta-analysis of randomised controlled trials with at least 12 months follow up. The results suggest that even with a very small intervention effect, such as an increase in fruit and vegetable consumption of half a portion per day, when maintained over prolonged periods of time might bring appreciable benefits in terms of number of years lived free from chronic disease. However, even when delivered at very low cost, we estimate that a universal intervention strategy is unlikely to prove cost-effective. We initially considered the possibility that the intervention could be delivered through resource use equivalent to one family practice consultation per year. However, our systematic review revealed that intervention strategies are generally more costly than this, requiring multiple face to face visits, singly or in groups, together with supporting written materials and follow-up communications. An effective intervention may be more costly and even less cost-effective that we have estimated. Our analysis suggests that a targeted strategy focusing on older individuals who may be at higher risk of disease may be a more appropriate strategy for implementation in primary care. However, more research is needed to explore the optimal strategy for targeting intervention. The results also serve to emphasise the importance of developing more effective interventions to promote dietary behaviour change.

### What other study show

Our conclusions are consistent with those of Cobiac et al. [32] who reviewed published literature and modelled the evidence available from range of 23 interventions for promoting fruit and vegetable consumption in adults. Their study concluded that interventions directed at individuals and relying on dietary counselling, telephone contact, worksite promotion and other methods which encourage dietary behaviour change are neither highly effective nor cost effective. Pomerlau et al. [33] also found that dietary change interventions generally had small effects, with larger effect sizes observed in secondary prevention studies. This is also consistent with the suggestion that a targeted intervention strategy may be more appropriate in primary care.

### Strengths and limitation of this study

This study was informed by the results of a new systematic review that was tailored to the objective of this research. The model was informed by analysis of a sample of nearly 300,000 participants sampled from the CPRD which is grounded in a general population sample of nearly six million people in the UK. The modelling approach was fully probabilistic which meant that uncertainties in the model inputs were carried through into uncertainties in the model outputs. This was necessary because estimates for incidence and mortality were imprecise for less frequent conditions. We implemented a series of sensitivity analyses that explored the effect of varying key assumptions. We acknowledge that we modelled an intervention duration (five years) that is longer than has been implemented in randomised trials (one year). However, it appears unlikely that a behavioural intervention could be more effective over a longer period than a shorter period, so this does not vitiate our conclusion that a universal intervention strategy in primary care is unlikely to be cost-effective. We focused on the effect of intervention on fruit and vegetable consumption but other dietary changes, such as consumption of fat or fibre, may also be associated with health outcomes. However, self-reported dietary change measures, used in many intervention studies, may have over-estimated the potential effects of intervention. A previous systematic review [18], found a larger intervention effect that but this review included high-risk groups including those with hypertension, hyperlipidaemia or having close relatives with type 2 diabetes or cancer. We also acknowledge that we did not utilise empirical data for the unit costs of intervention. We used a low estimate for the unit cost of intervention and, even with this estimate, the intervention strategy was found not to be cost-effective. More resource intensive interventions are likely to be even less cost-effective, as evidenced by the sensitivity analysis in which a higher intervention cost was used. Our systematic review [MS resubmitted to BMC Public Health] provides information on the resources used in previous intervention studies, and these interventions were generally considerably more resource intensive than the one modelled here. We did not model any social multiplier effects, where the impact of the intervention delivered to one person might motivate the individual’s social contacts to change. It is possible this may have underestimated the benefits of intervention. We did not set an upper age limit for participants as fruit and vegetable intake may be beneficial even in old age [34].We assessed the cost effectiveness of intervention in selected age groups of population which shows that intervention is more cost effective in older age groups. We applied a half cycle correction but this had only a small effect as the perspective of the model, up to 70 years, was long in relation to the length of the annual cycles. However, we acknowledge that there are arguments both for and against the implementation of a half-cycle correction [35,36]. We included only the health care costs; outcomes might differ if costs and productivity from a wider perspective were to be included. We obtained data from primary care records and we could not follow patient use of secondary care resources beyond referral and admission; this may have underestimated the utilisation of secondary care. We used the average of all costs over all stages of disease which may not have represented the actual resource utilisation costs as health care resource utilisation costs may be higher at the start of illness or at periods closer to death. Utility estimates were drawn from a compendium of values, which may not have provided an entirely accurate assessment for this population. We used a cost-effectiveness threshold of £30,000 per QALY as this is a widely accepted level below which interventions are judged to be cost-effective in the United Kingdom. However, some policy decisions have indicated that higher threshold values might sometimes be accepted in practice [37].

## Conclusions

Our findings provide insights into the potential for a universal strategy of brief intervention in primary care to promote dietary change. The expected dietary behavioural change from such a strategy is small, but even a modest increase in consumption of fruit and vegetables, may increase the number of life years lived free from physical disease and reduce the duration of time lived with chronic disease. The present results suggest that, even when implemented at very low unit cost, it is unlikely that a universal brief intervention strategy might have acceptable cost-effectiveness. There may be greater potential for a targeted strategy but further research is needed to identify appropriate strategies for targeting interventions. The main reason for the lack of cost-effectiveness is the very limited effects from intervention demonstrated in published intervention studies. There is therefore considerable scope to develop and implement more effective behavioural intervention strategies for dietary change.

### Ethics

The use of fully anonymised CPRD data was approved by the MHRA Independent Scientific Advisory Committee (Ref. 09-085).

## Competing interests

The authors declare that they have no competing interests.

## Authors’ contributions

CR and MCG designed the study; NB completed the systematic review that informed the analysis and wrote the first draft of the paper; JC programmed the Model and analysed the CPRD data; MCG implemented and analysed the simulations; CR contributed all health economic evaluation expertise; all authors critiqued and approved the final version of the manuscript.
